# Reconstruction of the Talus and Calcaneus Using a Three-Dimensional (3D)-Printed Custom Implant Following a Shotgun Wound: A Case Report

**DOI:** 10.7759/cureus.55723

**Published:** 2024-03-07

**Authors:** Christian Guevara, Samantha Trynz, Dino Fanfan, Cary Chapman

**Affiliations:** 1 Department of Orthopedic Surgery, Florida International University, Herbert Wertheim College of Medicine, Miami, USA; 2 Department of Orthopedic Surgery, Baptist Health South Florida, Miami, USA

**Keywords:** firearm injury, osteomyelitis, custom implants, foot and ankle trauma, three-dimensional (3d) printing

## Abstract

We present a case detailing the successful reconstruction of the hindfoot in a 15-year-old male patient who suffered a self-inflicted shotgun wound. The patient had multiple complex fractures in these bones, resulting in considerable bone loss and the destruction of the articular surface. Considering the extent of the injuries and the failure of prior intervention from an outside surgeon, traditional reconstruction methods would not have adequately addressed the severity of the damage. Consequently, the treating physician opted to address the deformity using a three-dimensional (3D)-printed custom implant to salvage the limb. The treatment involved a two-stage surgical plan. The first stage encompassed debridement with the removal of antibiotic cement, which had been placed at the time of the initial injury, followed by debridement and placement of a new temporary antibiotic spacer. A 21-day course of antibiotics was administered to combat the developing osteomyelitis. Following the successful eradication of the infection, a second surgery entailed removing the spacer and residual bone, inserting the 3D-printed implant filled with bone graft, and fusing the hindfoot. Post-surgery, the patient steadily progressed from non-weight-bearing to full weight-bearing and was fully weight-bearing at five months post-surgery. He had reported significant improvements in pain and mobility. There were no complications, and the 3D-printed implant exhibited excellent integration with the surrounding bone tissue with a two-year follow-up. This case serves as a demonstration of the utility of 3D-printed custom implants in severe foot and ankle trauma, showcasing the technology's potential to revolutionize orthopedic surgery. Despite the potential risks, this approach highlights significant benefits and opens avenues for tailored reconstructions in complex orthopedic injuries.

## Introduction

Foot and ankle trauma is a frequent cause of morbidity and disability [1]. In the event of severe and complex injuries, such as in the case of gunshot-related injuries, traditional surgical techniques may not provide adequate functional and structural reconstruction, which can lead to chronic pain and reduced mobility [2]. In complex hindfoot trauma, techniques for limb salvage involve the early reduction of existing fragments, fixation, and then, later, a subtalar joint arthrodesis when the limb is salvageable. Unfortunately, these types of trauma often present with extensive soft tissue injury, and this combination of factors makes biological reconstruction difficult and frequently unsuccessful [3].

Advances in three-dimensional (3D) printing technology have provided new opportunities for the creation of custom implants that can be tailored to the individual needs of patients [4,5]. These patient-specific constructs offer immediate support and function by creating precise anatomical models that facilitate accurate implant placement and potentially reduce complications, such as aseptic loosening or soft tissue irritation [5]. Moreover, they overcome limitations of allograft or autograft bone by limiting invasive tissue collection or avoiding possible disease transmission, altogether lowering donor-site morbidity [6-8].

This case report aims to demonstrate the successful use of a 3D-printed custom implant for the reconstruction of the talus and calcaneus following a gunshot wound.

## Case presentation

Indication

A 15-year-old male presented to our institution with a history of a self-inflicted shotgun wound to the right hindfoot two months prior. The patient had sustained highly comminuted fractures of the talus and calcaneus, resulting in significant bone loss and displacement of fragments. He underwent several surgeries at an outside facility, including initial debridement, antibiotic cement spacer placement, limb stabilization with an external fixator, placement of a right anterior lateral thigh free flap for coverage of the medial wound, and a lateral propeller flap for the lateral side of the foot. The patient was referred to our institution for a second opinion on bone grafting for limb salvage because of his bone loss. The patient and his family opted to proceed with limb salvage using a 3D-printed custom implant.

**Figure 1:**
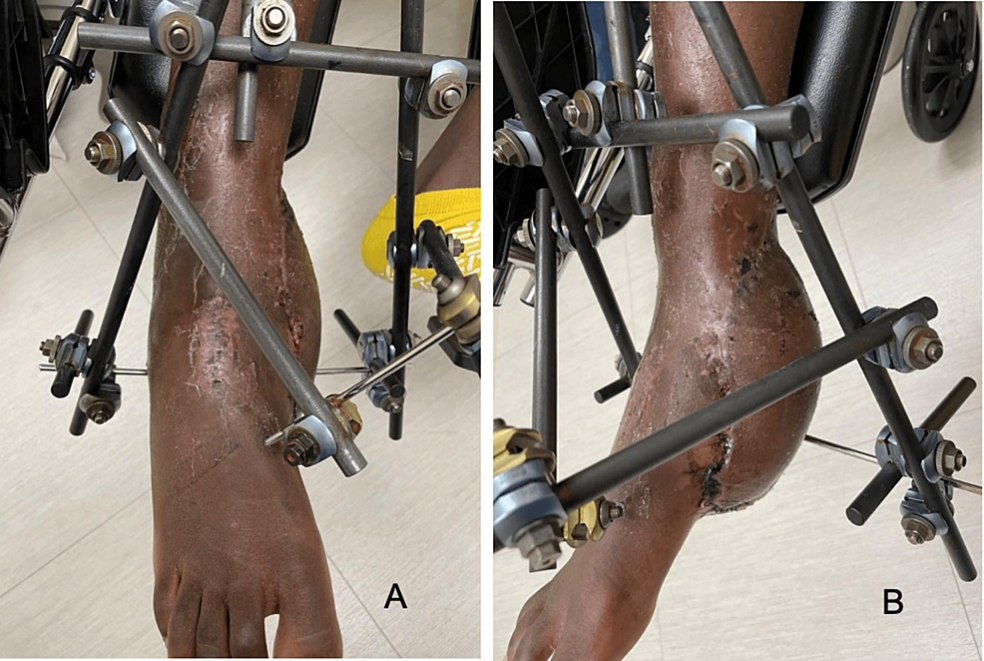
Wound with an external fixator in place. A: Anterior view of the medial wound with an external fixator in place. B: Medial view of the medial wound with an external fixator.

The patient presented to us as non-weight-bearing with an external fixator in place on the right lower extremity. A physical exam revealed a healed perforator-based propeller flap on the lateral ankle foot and an anterolateral thigh free-flap over the medial ankle (Figure 1).

**Figure 2:**
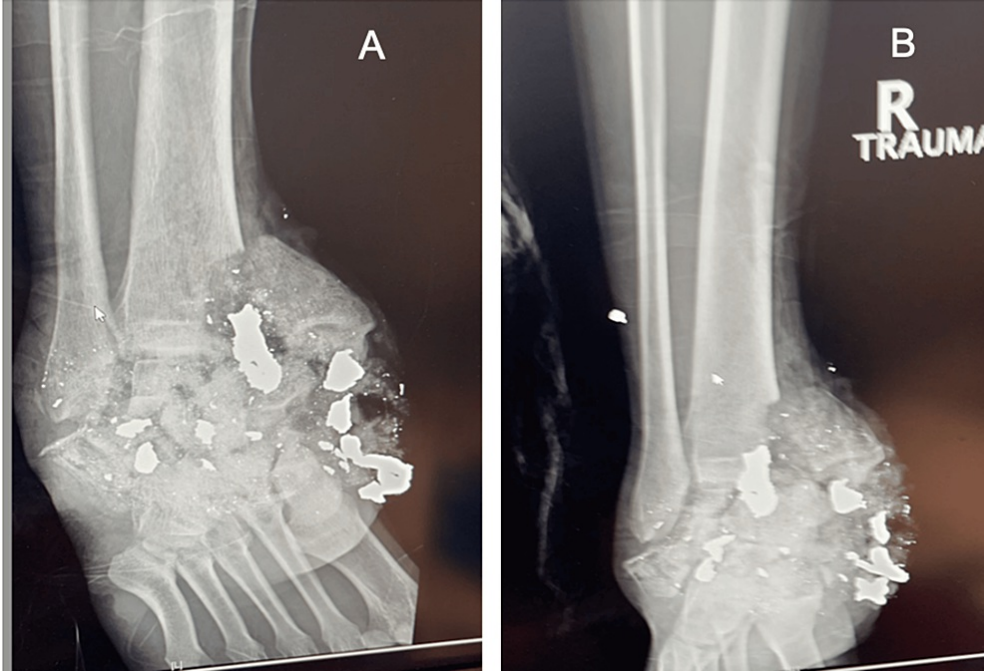
Preoperative radiographs of the injury. A: Oblique X-ray of the shotgun injury. B: Anterior-posterior X-ray from the shotgun injury.

Both flaps appeared to be healing well. Following a thorough clinical evaluation and radiographic images (Figure 2), the patient was considered a candidate for the reconstruction of the talus and calcaneus using a 3D-printed custom implant. A detailed 3D image of the patient's foot was obtained using computed tomography (CT) scans of the contralateral foot, which were then used to create a custom implant tailored to the patient's unique anatomy.

Surgical technique

Stage One

**Figure 3:**
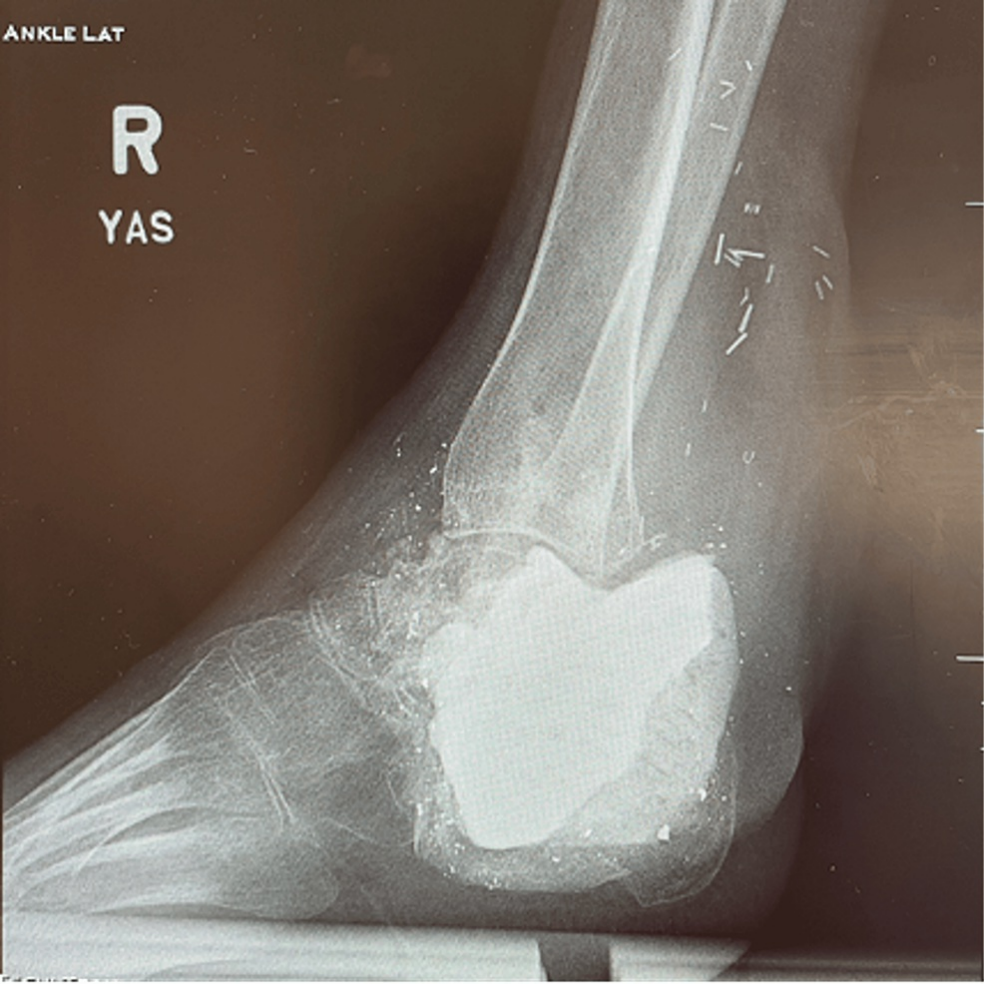
Radiograph after completion of stage 1 with antibiotic cement filler.

To access the ankle and remove the cement, a medial approach was taken, and the medial free flap was elevated. The wound could not be accessed anteriorly because of a tenuous skin bridge. The plastic surgeon concluded that using a straight anterior approach would create an incision in a very narrow bipedicle flap and recommended instead a medial approach through the previous incisions of the free flap. After the removal of the external fixator, an incision was made over the previous surgical scars, and the inferior 50% of the skin flap was elevated to allow for exposure of the previous antibiotic spacer while still maintaining the blood supply to the flap. Once exposed, cultures were taken, and the antibiotic-impregnated cement filler was removed. Additional deep cultures were also taken, and the remaining calcaneus and talus were debrided of any fibrotic bone. A new antibiotic-impregnated cement spacer was prepared with tobramycin, and vancomycin and was placed as a single block as a filler (Figure 3).

Cultures returned positive for staph species, and the patient was referred to an infectious disease specialist. The patient had no systemic symptoms, a normal white blood cell count, and normal inflammatory markers. However, the infectious disease team decided to treat the patient’s osteomyelitis with oral linezolid twice daily for 21 days. The surgical team decided that the patient should undergo one more washout of the wound and another round of cultures to ensure the eradication of the infection.

After the second irrigation and debridement, repeat cultures returned negative. Over the next four months, the patient remained non-weight-bearing in a boot, while a 3D implant was created. The implant was custom-made for this case from CT-guided measurements and was made of titanium with a titanium nitride coating.

Stage Two

**Figure 4:**
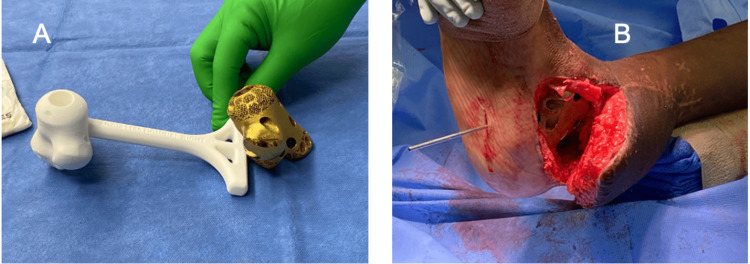
Implantation of the custom 3D implant. A: A 3D implant before implantation into the patient's foot. B. Intraoperative gross image after implantation.

This operation began with the plastic surgeon reopening the previous incision and elevating the skin flap in an atraumatic fashion with the maintenance of its blood supply. Once exposed, the antibiotic-impregnated cement was removed, and the residual talar head bone was excised. There appeared to be some contracture of the posterior ankle; thus, a posterior capsular release was performed, including Achilles tendon lengthening. The medial malleolus was removed to get the 3D-printed implant into the space (Figure 4).

The patient's own cancellous bone and bone marrow were taken from the proximal tibia for bone graft. The 3D-printed implant was packed with a combination of demineralized allograft bone powder mixed with autograft bone harvested from the proximal tibia. To begin the hindfoot fusion, a small incision was made on the plantar aspect of the foot, and a guidewire for the hindfoot nail was placed. An intramedullary hindfoot nail was then placed through the implant into the tibial shaft and secured with one distal locking screw. Two additional distal screws were placed, and the implant was compressed. The proper position of the construct was confirmed under fluoroscopic guidance. Multiple screws were then placed through the implant to the adjacent bone to perform a subtalar, tibiotalar, and calcaneocuboid fusion.

**Figure 5:**
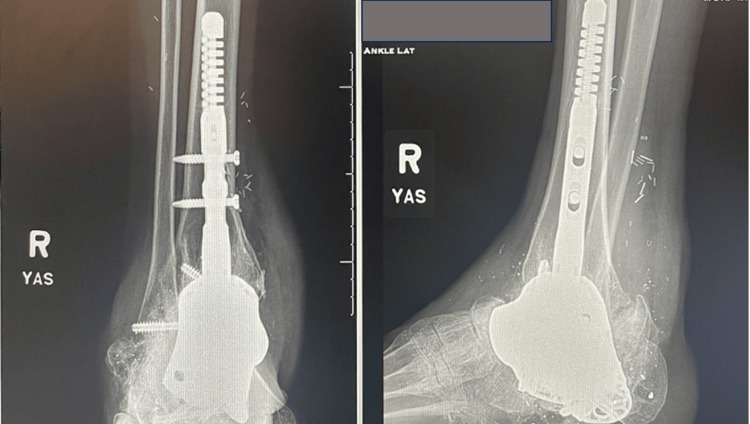
Anteroposterior and lateral radiographs at 12 months postoperatively showing a full union of the implant.

The patient was instructed to remain non-weight-bearing in a short leg cast using a bone stimulator. At the four-month follow-up visit, the patient began weight-bearing as tolerated in a cam boot and was fully weight-bearing within one month. At the one-year follow-up, the patient’s radiographs demonstrated full healing status post right hindfoot fusion and reconstruction with a 3D implant and intramedullary nail (Figure 5).

The patient was fully weight-bearing in a regular shoe. The patient had no complaints of pain other than some knee discomfort secondary to a slight plantar flexion deformity, which was corrected with a 1 cm heel lift.

## Discussion

The use of 3D printing technology for the creation of custom implants has revolutionized the field of orthopedic surgery [3,4]. In cases of severe and complex foot and ankle injuries, traditional surgical techniques may not provide adequate functional and structural reconstruction, leading to chronic pain and reduced mobility [2]. The case presented here illustrates the successful use of a 3D-printed custom implant for the reconstruction of the talus and calcaneus following a gunshot wound. The patient opted for limb salvage surgery after being presented with the options of hindfoot reconstruction with a 3D-printed implant or amputation.

The use of 3D printing technology allowed for the creation of an implant that was tailored to the unique pathology anatomy of the patient, allowing for better functional and structural reconstruction [4]. This technology has been used previously in the field of orthopedic surgery, with good results reported in several studies [9,10]. For example, Luenam et al. reported successful treatment of a radial head for chronic persistent elbow instability [9]. Similarly, Di Laura et al. described the use of 3D-printed custom implants for the reconstruction of complex acetabular defects, with good results [10]. These studies highlight the potential of 3D printing technology in the field of orthopedic surgery.

The use of 3D printing technology in the present case allowed for precise reconstruction of the talus and calcaneus, leading to good functional and structural outcomes. The 3D-printed implant was able to fill the bone defects left by the gunshot injury, providing stability and support to the foot. The patient could bear weight on the affected foot postoperatively and reported significant improvement in pain and mobility. The success of this case demonstrates the potential of 3D printing technology in the reconstruction of severe foot and ankle injuries.

It is important to note that the use of 3D-printed custom implants is not without potential complications. Infection is a potential risk associated with the use of any implant, and 3D-printed implants are no exception. In the present case, the patient had an infection because of the initial injury. It was essential to successfully treat the infection before the placement of the 3D implant.

## Conclusions

The present case illustrates the successful use of a 3D-printed custom implant for the reconstruction of the talus and calcaneus following a shotgun wound. This technology has the potential to revolutionize the field of foot and ankle orthopedic surgery, allowing for precise and tailored reconstruction of severe and complex injuries. While the use of 3D printing technology is not without potential complications, the success of this case highlights the potential benefits of this approach in the field of orthopedic surgery.

## Appendices

Statement of Informed Consent

The patient and his parents signed an informed consent form where they understood that his participation and the permission to use medical information is voluntary and he is free to withdraw at any time, without giving a reason and without a cost. A copy of the informed consent form was provided to the patient and his parents.
